# Does intraoperative closed-suction drainage influence the rate of pancreatic fistula after pancreaticoduodenectomy?

**DOI:** 10.1186/s12893-017-0257-3

**Published:** 2017-05-16

**Authors:** Ophélie Aumont, Aurélien Dupré, Adeline Abjean, Bruno Pereira, Julie Veziant, Bertrand Le Roy, Denis Pezet, Emmanuel Buc, Johan Gagnière

**Affiliations:** 1 Department of Digestive and Hepatobiliary Surgery, Estaing University Hospital, 1, place Lucie et Raymond Aubrac, 63000 Clermont-Ferrand, France; 2 Department of Surgical Oncology, Léon Bérard Cancer Center, Lyon, France; 3 Biostatistics, Délégation à la Recherche Clinique et à l’Innovation, University Hospital of Clermont-Ferrand, Clermont-Ferrand, France; 4 UMR 1071 INSERM / Clermont Auvergne University, Clermont-Ferrand, France

**Keywords:** Pancreaticoduodenectomy, Drainage, Pancreatic Fistula, Morbidity, Complications

## Abstract

**Background:**

Although drainage of pancreatic anastomoses after pancreaticoduodenectomy (PD) is still debated, it remains recommended, especially in patients with a high risk of post-operative pancreatic fistula (POPF). Modalities of drainage of pancreatic anastomoses, especially the use of passive (PAD) or closed-suction (CSD) drains, and their impact on surgical outcomes, have been poorly studied. The aim was to compare CSD versus PAD on surgical outcomes after PD.

**Methods:**

Retrospective analysis of 197 consecutive patients who underwent a standardized PD at two tertiary centers between March 2012 and April 2015. Patients with PAD (*n* = 132) or CSD (*n* = 65) were compared.

**Results:**

There was no significant difference in terms of 30-day overall and severe post-operative morbidity, post-operative hemorrhage, post-operative intra-abdominal fluid collections, 90-day post-operative mortality and mean length of hospital stay. The rate of POPF was significantly increased in the CSD group (47.7% vs. 32.6%; *p* = 0.04). CSD was associated with an increase of grade A POPF (21.5% vs. 8.3%; *p* = 0.03), while clinically relevant POPF were not impacted. In patients with grade A POPF, the rate of undrained intra-abdominal fluid collections was increased in the PAD group (46.1% vs. 21.4%; *p* = 0.18). After multivariate analysis, CSD was an independent factor associated with an increased rate of POPF (OR = 2.43; *p* = 0.012).

**Conclusions:**

There was no strongly relevant difference in terms of surgical outcomes between PAD or CSD of pancreatic anastomoses after PD, but CSD may help to decrease the rate of undrained post-operative intra-abdominal collections in some patients. Further randomized, multi-institutional studies are needed.

## Background

Despite recent improvements in surgical techniques and peri-operative management, the post-operative morbidity after pancreaticoduodenectomy (PD) remains high, ranging from 16 to 77% [1–12]. Post-operative pancreatic fistula (POPF) is the most frequent and feared complication after PD, reported in 5 to 48% of patients [2–5, 7, 9, 10, 12, 13] and is responsible for a high post-operative mortality that could reach 12% after PD [2–5, 7, 9]. POPF is also linked to other post-operative complications, such as delayed gastric emptying and hemorrhage, which can extend the length of hospital stay, increase the readmission rate and raise health care costs. Moreover, POPF could be responsible for delayed adjuvant chemotherapy administration that could alter the prognosis of patients treated for pancreatic cancer [7, 14–19]. Preventive strategies such as main pancreatic duct (MPD) drainage, the use of somatostatin analogs or biological sealants, and the optimization of pancreatic anastomosis techniques have failed to decrease significantly the rate of POPF after PD.

Drainage of the pancreatic anastomosis is routinely used after PD to allow earlier diagnosis of POPF and to prevent/diagnose its related complications, especially hemorrhages. However, drainage of the operative site could be responsible of a specific post-operative morbidity, particularly infectious complications, post-operative pain and increased lengths of hospital stay [20–24]. Although prophylactic drainage in hepatic and colorectal surgery has shown no clear benefit on post-operative morbidity [25–28], the problematic is highly different in pancreatic surgery due to the high risk of non-diagnosed and undrained POPF, with high risk of post-operative hemorrhage and death. On the other hand, drain can increase post-operative morbidity through the increase of surgical site infection. Routine drainage in pancreatic surgery remains controversial [11, 24, 29–34], but several studies have reported a significant increase of both morbidity and mortality in the absence of drainage [11, 30, 35]. Thus, regarding the current literature, drainage of pancreatic anastomoses after PD remains still recommended, especially in patients with a high risk of POPF [36], and should therefore be optimized. Indeed, modalities of drainage of pancreatic anastomoses, especially the use of passive (PAD) or closed-suction (CSD) drains, strongly vary among surgical teams, and their impact on surgical outcomes has been poorly studied. The aim of our study was to compare the use of CSD versus PAD on surgical outcomes after PD at two tertiary centers.

## Methods

### Study population and data collection

We retrospectively analyzed data from all patients who underwent a PD for benign or malignant tumors of the head of the pancreas or peri-ampullary area at two tertiary centers between March 2012 and April 2015. The recorded data included the patient demographics, co-morbidities, ASA score, need for pre-operative biliary drainage, administration of a neoadjuvant treatment, intra-operative blood loss, blood transfusions, operative time, intra-operative MPD diameter and pancreatic gland texture, need for vascular resection, type of pancreatic anastomosis, pylorus preservation, modality of drainage of the pancreatic anastomosis and/or the MPD, amylase levels on operatively placed drains, use of somatostatin analogs, type and severity of post-operative complications, length of hospital stay and histopathological data.

### Treatment and follow-up

Therapeutic management for all patients was systematically discussed in digestive cancer board meetings at our institutions. A standardized classical Whipple procedure was usually performed. Reconstruction with duct-to-mucosa pancreaticojenunostomy or pancreaticogastrostomy was at the surgeon’s discretion, with routine use of external drainage of the MPD. Sealants were not used. At the end of the procedure, one PAD or CSD (Shirley drain) was systematically placed near the pancreatic anastomosis at surgeon’s discretion in both centers.

All of the patients were treated by a standardized post-operative care pathway for pancreatic resection used at our institutions. Patients were systematically transferred to an intensive care unit post-operatively. Somatostatin analogs were used at the operating surgeon’s discretion. Drain outputs were recorded, and amylase levels were measured at post-operative day 1, 3 and 5. Abdominal drains were removed at post-operative day 5, except in case of POPF or biliary leak. A computed tomography (CT)-scan was systematically performed at post-operative day 5, to detect undrained intra-abdominal collections that were systematically drained with percutaneous CT-guided and/or endoscopic trans-gastric and/or surgical approaches. Amylase levels in post-operatively drained intra-abdominal collections were also systematically measured to detect POPF. Additional management methods for suspected POPF included the administration of antibiotics and supplemental parenteral or enteral nutrition support. Every post-operative complications occurring during the first 90 post-operative days were recorded.

### Endpoints and definitions

Endpoints included both rate and severity of POPF (according to the ISGPF definition and classification [37]), post-operative hemorrhage and intra-abdominal fluid collections rates, 30-day overall post-operative morbidity rate and severity (according to the Dindo and Clavien classification [38]), 90-day post-operative mortality and the mean length of hospital stay.

### Statistical analysis

All analyses were performed using Stata software (version 13; StataCorp, College Station, TX) and were performed for a two-sided type I error of α = 5%. Baseline characteristics were presented as the mean ± standard deviation for continuous data (Shapiro-Wilk test was used to assess normality), and as the number of patients and associated percentages for categorical parameters. Comparisons of the patient’s characteristics between groups were carried out using the chi-squared test for categorical variables, and Student’s t-test or the Mann-Whitney test when assumptions of the t-test were not met (normality and homoscedasticity studied using Fisher-Snedecor test) for quantitative variables. Next, a generalized linear regression model (logistic for dichotomous dependent outcome) was considered to study the effect of predictive factors in multivariate analysis by backward and forward stepwise regression of the factors considered significant in univariate analysis and according to clinically relevant parameters. The results were expressed as odds ratios (OR) and 95% confidence intervals. The final model was analyzed by a two-step bootstrapping process.

## Results

One hundred and ninety-seven consecutive patients underwent PD for benign or malignant tumors involving the head of the pancreas or the peri-ampullary area at two tertiary centers between March 2012 and April 2015. The clinicopathological and therapeutic features are detailed in Table 1.

**Table 1:** Clinicopathological and therapeutic features of patients who underwent duodenopancreatectomy

Characteristics	Study population (*n* = 197)		Passive drainage (*n* = 132)		Closed-suction drainage (*n* = 65)		
	No./mean ± SD^a^	(%)	No./mean ± SD^a^	(%)	No./mean ± SD^a^	(%)	*p*
Age (years)	66.2 ± 11.8	-	64.8 ± 11.3	-	68.5 ± 11.8	-	0.08
Male	108	(54.8)	70	(53.0)	38	(58.5)	0.47
Body mass index (kg/m^2^)	25.0 ± 4.3	-	24.7 ± 4.1	-	25.5 ± 4.4	-	0.21
ASA^b^ score							0.31
1	44	(22.3)	34	(25.0)	10	(15.4)	
2	114	(57.9)	75	(56.8)	39	(60.0)	
3	39	(18.3)	23	(17.4)	16	(24.6)	
Chronic pancreatitis	30	(19.8)	19	(14.4)	12	(18.5)	0.46
Pathology							<0.01
Adenocarcinoma	134	(68.0)	97	(73.5)	37	(56.9)	
Cholangiocarcinoma	7	(3.6)	6	(4.5)	1	(1.5)	
Ampullocarcinoma	6	(3.0)	5	(3.8)	1	(1.5)	
Other	50	(25.4)	24	(18.2)	26	(40.1)	
Neoadjuvant treatment	22	(11.2)	13	(9.8)	9	(13.8)	0.40
Pre-operative biliary drainage	93	(47.2)	66	(50.0)	27	(41.5)	0.26
Artery resection	1	(0.5)	1	(0.8)	0	(0)	1.00
Vein resection	38	(19.3)	32	(24.2)	6	(9.2)	0.01
Pylorus preservation	142	(72.1)	106	(80.3)	36	(55.4)	<0.01
Type of pancreatic anastomosis							<0.01
Pancreaticogastrostomy	97	(49.2)	79	(60.3)	18	(28.1)	
Pancreaticojejunostomy	98	(49.8)	52	(39.7)	46	(71.9)	
Soft pancreatic gland texture	28	(14.2)	18	(13.6)	10	(15.4)	0.66
Main pancreatic duct diameter	4.93 ± 2.45	-	5.0 ± 2.3	-	5.1 ± 2.7	-	0.79
Intra-operative blood loss (mL)	685 ± 588	-	664 ± 519	-	728 ± 719	-	0.89
Blood transfusion	44	(22.3)	28	(22.8)	16	(24.6)	0.78
Operative time (minutes)	388 ± 125	-	339 ± 136	-	377 ± 95	-	0.16
Somatostatin analogs	89	45.2	61	47.3	28	43.7	0.65

^a^Standard deviation

^b^American Society of Anesthesiology

Patients with PAD (*n* = 132) or CSD (*n* = 65) were compared (Table 1). Pylorus-preserving PD (80.3% vs. 55.4%; *p* = 0.001), venous resections (24.2% vs. 9.2%; *p* = 0.012), pancreaticogastrostomies (60.3% vs. 28.1%; *p* = 0.001) and adenocarcinomas (73.5% vs. 56.9%; *p* = 0.008) were preferentially reported in patients with PAD. Both groups were comparable regarding BMI (*p* = 0.21), chronic pancreatitis rates (*p* = 0.46), operative time (*p* = 0.16), intraoperative blood loss (*p* = 0.89), pancreas texture (*p* = 0.66), drainage of the MPD (*p* = 0.79), and use of somatostatin analogs (*p* = 0.65).

There was no significant difference between the two groups in terms of 30-day overall (71.2% vs. 70.8% (*n* = 46); *p* = 0.93) and severe (grade ≥ III) (37.9% vs. 40%; *p* = 0.93) post-operative morbidity, post-operative hemorrhage (9.0% vs. 9.2%; *p* = 0.97), 90-day post-operative mortality (15.2% vs. 12.3%; *p* = 0.59) and mean length of hospital stay (28 ± 18 days vs. 31 ± 48 days; *p* = 0.17) (Table 2).

**Table 2:** Studied endpoints of patients who underwent duodenopancreatectomy

Endpoints	Study population (*n* = 197)		Passive drainage (*n* = 132)		Closed-suction drainage (*n* = 65)		
	No./mean ± SD^a^	(%)	No./mean ± SD^a^	(%)	No./mean ± SD^a^	(%)	*p*
30-day post-operative morbidity							0.93
Overall	140	(71.0)	94	(71.2)	46	(70.8)	
Grade I-II	64	(32.5)	44	(33.3)	20	(30.8)	
Grade ≥ III	76	(38.6)	50	(37.9)	26	(40.0)	
POPF^b^							
Overall	75	(38.1)	43	(32.6)	32	(47.7)	0.04
Grade A	25	(12.7)	11	(8.3)	14	(21.5)	0.03
Grade B	22	(11.2)	14	(10.6)	8	(12.3)	0.74
Grade C	27	(13.7)	18	(13.6)	9	(13.8)	0.91
Intra-abdominal fluid collection	45	(22.9)	28	(21.1)	17	(26.1)	0.44
Post-operative hemorrhage	18	(9.1)	12	(9.0)	6	(9.2)	0.97
90-day post-operative mortality	28	(14.2)	20	(15.2)	8	(12.3)	0.59
Length of hospital stay (days)	30 ± 33	-	28 ± 18	-	31 ± 48	-	0.17

^a^Standard deviation

^b^Post-operative pancreatic fistula

The rate of POPF was significantly increased in the CSD group (47.7% vs. 32.6%; *p* = 0.04). Considering the grade of POPF according to the ISGPF classification, CSD was associated with an increase of grade A POPF (21.5% vs. 8.3%; *p* = 0.03), while clinically relevant grade B/C POPF were not influenced by the modality of drainage (Table 2).

Given the fact that the higher rate of grade A POPF in the CSD group could be explained by a more accurate diagnosis of latent POPF, we investigated the rate of undrained post-operative intra-abdominal fluid collections between the two groups. This rate was not significantly different between the two groups (21.1% in the CSD group vs. 26.1% in the PAD group; *p* = 0.44) (Table 2). In patients with grade A POPF (*n* = 27), the rate of undrained intra-abdominal fluid collections was increased in the PAD group (46.1% vs. 21.4%) but this difference was not significant (*p* = 0.18). In patients with clinically relevant grade B/C POPF (*n* = 51), this rate was comparable between the two studied groups (47.0% vs. 58.8%; *p* = 0.43) (Table 2).

After multivariate analysis, only CSD was an independent factor associated with an increased rate of POPF after PD (OR = 2.43, 95% CI [1.21–4.87]; *p* = 0.012).

## Discussion

Systematic drainage after PD is still debated [11, 24, 29–35] but remains still recommended in patients with a high risk of POPF [36]. Optimization of the drainage modalities is a major concern, with the aim to improve detection of POPF and to prevent/diagnose related complications, especially hemorrhages. We present herein a study which has compared the efficacy of closed-suction drainage versus passive drainage of pancreatic anastomoses on surgical outcomes after PD. We reported that the rate of POPF, especially regarding grade A POPF, was significantly increased in patients with CSD, but there was no impact on clinically relevant grade B/C POPF, post-operative morbidity and length of hospital stay. Nevertheless, it remains unclear whether CSD allows better detection of grade A POPF or could directly favor it. Patients in the PAD group with grade A POPF had more undrained peri-anastomotic intra-abdominal fluid collections. This could be explained by undiagnosed POPF according to the ISGPF definition [37] and a higher efficacy of CSD. However, as grade A POPF do not imply any specific therapeutic changes, and as grade B/C POPF were similar in both groups, we can conclude that there was no strongly relevant difference in terms of surgical outcomes between PAD and CSD, but that CSD could probably help to decrease rate of undrained post-operative intra-abdominal collections in some patients.

As mentioned above, the relation between the modalities of intra-operative drainage and the postoperative complications after PD has been poorly studied. The use of single or multiple drains in pancreatic surgery has failed to show any relevant interest in a single center retrospective study [39]. In the same topic as our study, a chinese randomized trial from Jiang et al. has reported a decrease of both grade C POPF rate and length of hospital stay with CSD [40]. However, even randomized, this study cannot provide adequate conclusions due to the presence of major biases. Firstly, the sample size was calculated based on the overall post-operative morbidity as primary endpoint, and did not thus allow making formal conclusions on secondary endpoints such as the rate of POPF. Secondly, no systematic post-operative CT-scan was performed, that could thus have underestimated both POPF (especially grade A POPF) and intra-abdominal fluid collections rates. And most importantly, the unusual use of CSD that were systematically irrigated with 3 l normal saline every day for the first 3 post-operative days and kept with intermittent suction for the following 2 days has to be highlighted. It is important to note that most of the studies that have investigated the role of drainage in pancreatic surgery reported the use of CSD [24, 29, 31–33]. Even if CSD were not compared to PAD in these studies, some reported no benefit of CSD over no drain [24, 33], while others reported less intra-abdominal undrained fluid collections with CSD [31]. Further specific studies are thus needed, such as the DRAPA trial [41].

Our study demonstrated a benefit of CSD over PAD, but only to detect grade A POPF, with no clinical relevance. However, this was limited by its retrospective nature and by imbalanced compared groups. Indeed, patients in the CSD group were older (*p* = 0.08), had significantly less adenocarcinomas (*p* < 0.01), and high operative time (+38 min; *p* = 0.16), which are known risks of relevant POPF. Furthermore, type of pancreatic anastomosis was different between the two groups, which could have introduced a bias. Thus, this work could constitute a basis for further controlled randomized, multi-institutional studies to validate these conclusions. Regarding emerging concepts of enhanced recovery which favors the absence or early removal of drains, these studies should especially include patients with high risk of POPF [42–44], who would probably benefit from the drainage of pancreatic anastomoses [36, 45].

## Conclusions

The rate of grade A POPF after PD was significantly increased in patients with CSD, but that modality of drainage did not impact clinically relevant grade B/C POPF, post-operative morbidity, mortality and length of hospital stay. The rate of undrained peri-anastomotic intra-abdominal fluid collections in patients carrying a PAD and presenting with grade A POPF was also increased. Regarding the fact that grade A POPF do not imply any specific therapeutic changes we can conclude that, if performed, there was no strongly relevant difference in terms of surgical outcomes between PAD or CSD after PD, but that CSD could probably help to decrease the rate of undrained post-operative intra-abdominal collections in some patients. Further randomized, multi-institutional studies are needed to validate these conclusions.
